# Optimization of the doxycycline-dependent simian immunodeficiency virus through in vitro evolution

**DOI:** 10.1186/1742-4690-5-44

**Published:** 2008-06-05

**Authors:** Atze T Das, Bep Klaver, Mireille Centlivre, Alex Harwig, Marcel Ooms, Mark Page, Neil Almond, Fang Yuan, Mike Piatak, Jeffrey D Lifson, Ben Berkhout

**Affiliations:** 1 Laboratory of Experimental Virology, Department of Medical Microbiology, Center for Infection and Immunity Amsterdam (CINIMA), Academic Medical Center of the University of Amsterdam, The Netherlands; 2 Division of Retrovirology, National Institute for Biological Standards and Control, Potters Bar, UK; 3 AIDS Vaccine Program, SAIC Frederick, Inc., National Cancer Institute at Frederick, Frederick, Maryland 21702, USA

## Abstract

**Background:**

Vaccination of macaques with live attenuated simian immunodeficiency virus (SIV) provides significant protection against the wild-type virus. The use of a live attenuated human immunodeficiency virus (HIV) as AIDS vaccine in humans is however considered unsafe because of the risk that the attenuated virus may accumulate genetic changes during persistence and evolve to a pathogenic variant. We earlier presented a conditionally live HIV-1 variant that replicates exclusively in the presence of doxycycline (dox). Replication of this vaccine strain can be limited to the time that is needed to provide full protection through transient dox administration. Since the effectiveness and safety of such a conditionally live virus vaccine should be tested in macaques, we constructed a similar dox-dependent SIV variant. The Tat-TAR transcription control mechanism in this virus was inactivated through mutation and functionally replaced by the dox-inducible Tet-On regulatory system. This SIV-rtTA variant replicated in a dox-dependent manner in T cell lines, but not as efficiently as the parental SIVmac239 strain. Since macaque studies will likely require an efficiently replicating variant, we set out to optimize SIV-rtTA through in vitro viral evolution.

**Results:**

Upon long-term culturing of SIV-rtTA, additional nucleotide substitutions were observed in TAR that affect the structure of this RNA element but that do not restore Tat binding. We demonstrate that the bulge and loop mutations that we had introduced in the TAR element of SIV-rtTA to inactivate the Tat-TAR mechanism, shifted the equilibrium between two alternative conformations of TAR. The additional TAR mutations observed in the evolved variants partially or completely restored this equilibrium, which suggests that the balance between the two TAR conformations is important for efficient viral replication. Moreover, SIV-rtTA acquired mutations in the U3 promoter region. We demonstrate that these TAR and U3 changes improve viral replication in T-cell lines and macaque peripheral blood mononuclear cells (PBMC) but do not affect dox-control.

**Conclusion:**

The dox-dependent SIV-rtTA variant was optimized by viral evolution, yielding variants that can be used to test the conditionally live virus vaccine approach and as a tool in SIV biology studies and vaccine research.

## Background

More than 20 years after the identification of human immunodeficiency virus (HIV) as the causative agent of AIDS, an effective HIV/AIDS vaccine remains elusive. All vaccine candidates thus far tested in human efficacy trials have failed to prevent HIV infection or suppress the viral load. In the experimental model system of pathogenic simian immunodeficiency virus (SIV) in macaques, live attenuated virus vaccines have proven to be much more effective than other AIDS vaccine approaches. For example, 95% of the Indian rhesus macaques immunized with a live attenuated SIV demonstrated a viral load suppression of more than 3 logs (compared to unvaccinated animals) upon challenge with a wild-type SIV, whereas such protection was observed in only 7% of macaques immunized with other vaccine strategies [1]. In most of the studies, SIV was attenuated through deletion of one or several accessory functions from the viral genome (reviewed in [1-4]). Although the majority of macaques vaccinated with such deletion variants of SIV can efficiently control replication of pathogenic challenge virus strains, the attenuated virus could revert to virulence and cause disease over time in some vaccinated animals [5-8]. Similarly, some of the long-term survivors of the Sydney Blood Bank Cohort infected with an attenuated HIV-1 variant in which nef and long terminal repeat (LTR) sequences were deleted, eventually progressed to AIDS [9]. An HIV-1 Δ3 variant with deletions in the vpr, nef and LTR sequences regained substantial replication capacity in long-term cell culture infections by acquiring compensatory changes in the viral genome [10]. These results underline the evolutionary capacity of attenuated SIV/HIV strains, which poses a serious safety risk for any future experimentation with live attenuated HIV vaccines in humans.

Evolution of the attenuated vaccine virus upon vaccination is due to the persistence of the virus and ongoing low-level replication. The error-prone viral replication machinery can facilitate the generation and accumulation of mutations in the viral genome that improve replication and pathogenicity. To minimize the prospect of such undesired evolution of the vaccine strain, we and others previously presented a unique genetic approach that exploits a conditionally live HIV-1 variant [11-15]. In our HIV-rtTA variant, the Tat-TAR regulatory mechanism that controls viral transcription was inactivated by mutation of both the Tat protein and the TAR RNA element, and functionally replaced by the components of the Tet-On system for inducible gene expression [16]. The rtTA gene encoding a synthetic transcriptional activator was inserted in place of the nef gene, and the corresponding tet-operator (tetO) DNA binding sites were inserted into the LTR promoter. Since the rtTA protein can only bind tetO and activate transcription in the presence of doxycycline (dox), HIV-rtTA replicates exclusively when dox is administered. Upon vaccination with this virus, replication can be switched on temporarily and controlled to the extent needed for induction of the immune system by transient dox administration. Upon long-term in vitro passage of the initial HIV-rtTA variant on T cells, the virus acquired additional modifications in both the rtTA and tetO components that significantly improved replication [17-22]. This designer HIV-rtTA was thus optimized through in vitro virus evolution, resulting in a dox-dependent variant that replicates in vitro in T cell lines and ex vivo in human lymphoid tissue [23]. In addition, we constructed an HIV-1 variant that depends not only on dox for gene expression, but also on the T20 peptide for cell entry [24].

To evaluate the safety and effectiveness of such a conditionally replicating virus as a candidate AIDS vaccine, a dox-dependent SIV variant is needed that can be tested in macaques. Moreover, such an SIV variant may be an ideal tool to study the immune correlates of vaccine protection, since both the level and duration of virus replication can in principle be controlled by dox administration. Such studies may reveal the critical information needed for the design of an HIV vaccine that is safe and equally effective as a live attenuated virus. Based on our experience in developing HIV-rtTA, we recently constructed a similar dox-dependent SIVmac239 variant [25]. Surprisingly, inactivation of the Tat protein was not allowed in the SIV-rtTA context, even though gene expression was transcriptionally controlled by the incorporated Tet-On system. This result suggests that Tat has additional essential functions in SIV replication in addition to its role in the activation of transcription. The Tat-positive SIV-rtTA variant replicated in a dox-dependent manner in T cell lines, but not as efficiently as the parental SIVmac239 strain. We anticipated that SIV-rtTA could evolve to a better replicating variant and therefore initiated multiple cultures. We did indeed identify modifications in the U3 and TAR regions that significantly enhance SIV-rtTA replication in T cell lines and macaque peripheral blood mononuclear cells (PBMC). Importantly, these modifications do not affect dox-control. These evolved SIV-rtTA variants should allow future in vivo studies in macaques.

## Results

### In vitro evolution of the dox-inducible SIV-rtTA variant

**Figure 1:**
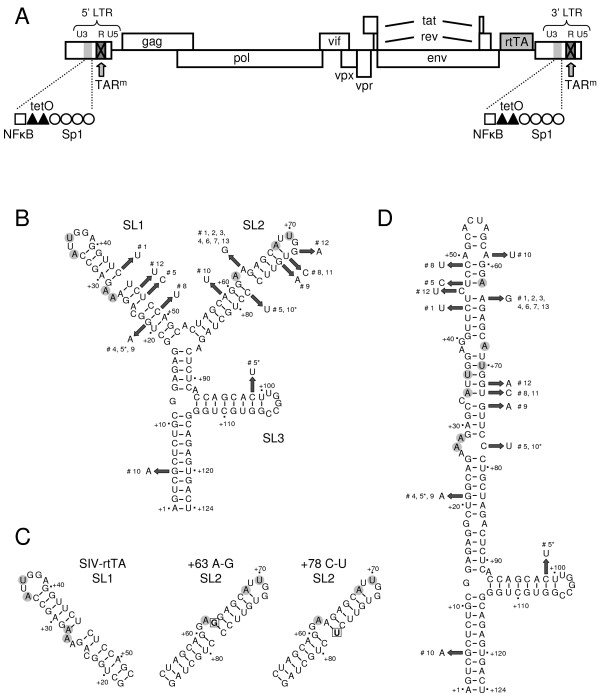
**Evolution of the dox-inducible SIV-rtTA variant**. (A) In the SIVmac239-based SIV-rtTA variant, the Tat-TAR regulatory mechanism was inactivated through mutation of TAR (TAR^m^), and functionally replaced by the dox-inducible Tet-On regulatory system through the introduction of the gene encoding the rtTA transcriptional activator protein at the site of the nef gene and two dox-responsive tet operator (tetO) elements between the NFκB and Sp1 sites in the U3 promoter region [25]. The TAR mutations and tetO elements were introduced in both the 5' and 3' LTR. (B) The TAR RNA element of SIV-rtTA can fold a branched hairpin structure with three stem-loop domains (SL1-3). The mutations that had been introduced in SL1 and SL2 to inactivate TAR, are encircled in gray (SL1: +27^U-A^, +28^U-A^, +34^C-A^, +36^G-U^; SL2: +62^U-A^, +68^C-A^, +70^G-U^). Upon long-term culturing of SIV-rtTA in PM1 cells, additional nucleotide substitutions are observed in TAR. The number of the culture in which the mutation is observed is shown (#), with the asterisk (*) indicating the transient presence of the mutation. (C) Alternative folding of the SL1 domain can result in a 6-bp spacer between the bulge and loop sequences. However, this spacer extension slightly reduces TAR stability (ΔG_5 bp _= -67.5 kcal/mole; ΔG_6 bp _= -67.2 kcal/mole). Alternative folding of the +63^A-G ^mutated TAR RNA results in a 6-bp bulge-loop spacer in SL2 but does not affect TAR stability (ΔG_5 bp _= ΔG_6 bp _= -67.5 kcal/mole). Formation of an A^+63^-U^+78 ^base pair in the +78^C-U ^mutant results in a similar 6-bp bulge-loop spacer in SL2 and increases the stability of this TAR variant (ΔG_5 bp _= -65.2 kcal/mole; ΔG_6 bp _= -65.8 kcal/mole). (D) TAR can fold an alternative extended hairpin structure in which the SL1 and SL2 sequences fold a large stem-loop structure. The introduced and acquired mutations are shown as in B.

We recently described the construction of a dox-dependent SIVmac239 variant in which the natural Tat-TAR mechanism of transcription control was replaced by the dox-inducible Tet-On gene expression system (Fig. 1A). In this variant, the bulge and loop sequences in stem-loop 1 (SL1) and stem-loop 2 (SL2) of TAR are mutated (TAR^m^; substituted nucleotides marked in a gray circle in Fig. 1B), which prevents the binding of Tat and precludes Tat-responsiveness of the LTR promoter. Furthermore, this virus carries the gene encoding the rtTA transcriptional activator protein at the position of the nef gene and two dox-responsive tet operator (tetO) elements between the NFκB and Sp1 binding sites in the U3 promoter region (Fig. 1A). Dox induces a conformational change in the rtTA protein that triggers binding to the tetO sites and activation of transcription from the downstream start site. In the absence of dox, rtTA cannot bind to the tetO sites and viral gene expression is not activated. Since transcription is critically dependent on dox, this SIV-rtTA variant replicates exclusively in the presence of dox. As the TAR mutations and tetO elements were introduced in both the 5' and 3' LTR, they are stably maintained in the viral progeny.

**Figure 2:**
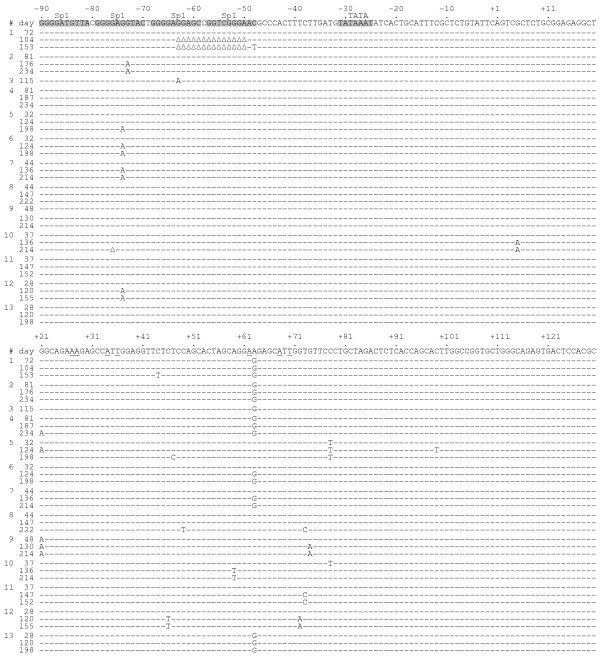
**SIV-rtTA acquires additional mutations in the U3 and TAR region upon long-term culturing**. SIV-rtTA was cultured with dox in PM1 cells for up to 234 days. Cellular proviral DNA was isolated from 13 independent cultures at different times and the LTR region was subsequently PCR amplified and sequenced. The number of the culture (#) and the day of sampling are indicated on the left. The -90 to +130 U3/R region is shown with +1 indicating the transcription initiation site. The Sp1 and TATA box are shown in grey. The mutations that were introduced in TAR to abolish Tat-responsiveness are underlined. The additional nucleotide substitutions and deletions (Δ) observed in the SIV-rtTA cultures are indicated.

We demonstrated that SIV-rtTA replicates in a dox-dependent manner in PM1 T-cells, but not as efficiently as the wild-type SIVmac239 variant [25]. Since macaque studies will likely require an efficiently replicating variant, we set out to optimize SIV-rtTA through in vitro viral evolution. We therefore started 13 cultures of the Tat-positive SIV-rtTA variant in PM1 cells and passaged the virus onto fresh cells at the peak of infection when massive syncytia were observed. The cultures were maintained for up to 234 days. The period between infection and the appearance of syncytia decreased over time and we could reduce the volume of the virus inoculum that is needed to start a new infection. These observations indicate that the replication capacity of the virus had improved and we analyzed the proviral genome present in these long-term cultures. This analysis revealed that the virus stably maintained the introduced TAR mutations, rtTA gene and tetO elements, but acquired additional mutations in the LTR region (Fig. 2). We observed one or several nucleotide substitutions in the TAR sequence in all 13 cultures. In eight of these cultures, additional nucleotide substitutions or deletions were present in the Sp1 sites, which are located between the tetO sites and the TATA box.

### Mutations in TAR affect RNA structure

We observed an A-to-G substitution at TAR position +63 in seven independent cultures (Fig. 1B). The high frequency may indicate that this change is an important evolutionary route toward improved replication. This substitution may induce a base pairing rearrangement in SL2 by formation of a G^+63^-C^+78 ^base pair, resulting in a 6-bp spacer between the bulge and loop domains (Fig. 1C). Remarkably, we observed a C-to-U substitution at position +78 in two other cultures that has the same impact on the TAR structure, as it also allows the formation of a 6-bp bulge-loop spacer through A^+63^-U^+78 ^base pairing in SL2 (Fig. 1C). In fact, the mutated SL1 can also form a 6-bp spacer between the bulge and loop domains (Fig. 1C), although analysis of the thermodynamic stability with the MFold RNA folding software [26,27] revealed that this spacer extension slightly reduces TAR stability (ΔG_5 bp _= -67.5 kcal/mole; ΔG_6 bp _= -67.2 kcal/mole). Another remarkable mutation is seen at position +21 in three cultures. This G-to-A mutation destabilizes the lower stem of SL1 by generating an A^+21^-C^+49 ^mismatch but it creates a 7-nt sequence (CUAGCAG) at the start of the SL1 sequence that is repeated at the start of SL2. Nearly all other nucleotide substitutions were observed in individual cultures. These mutations seem to destabilize the TAR structure by either replacing a G-C base pair by a less stable G-U base pair, or by causing a base pair mismatch (Fig. 1B).

Recently, Pachulska-Wieczorek et al. showed that HIV-2 TAR can fold an alternative secondary structure in addition to the classical branched hairpin (BH) structure with SL1, SL2 and SL3 [28]. In this extended hairpin (EH) structure, the SL1 and SL2 sequences fold a single, extended stem-loop structure. SIVmac239 TAR, which is very similar to HIV-2 TAR, and the mutated SIV-rtTA TAR may also co-exist in comparable BH and EH forms (Fig. 1B and 1D, respectively). At first glance, the individual TAR mutations observed in SIV-rtTA upon prolonged culturing seem to either stabilize the EH structure by creating more stable base pairs (e.g. replacement of a G-U base pair by a more stable A-U base pair) or destabilize this structure by creating mismatches or less stable base pairs (e.g. replacement of a G-C base pair by a G-U base pair). Since the equilibrium between the BH and EH conformers may be essential in viral replication, we used MFold RNA analysis to estimate the thermodynamic stability of the BH and EH structures for the wild-type (TAR^wt ^in SIVmac239), mutated (TAR^m ^in SIV-rtTA) and evolved TAR sequences (Table 1). The difference between these ΔG values (ΔΔG_BH-EH_) reflects whether the BH form is more stable and favored (ΔΔG_BH-EH _< 0) or the EH form (ΔΔG_BH-EH _> 0). This analysis revealed that TAR^wt ^is more stable in the EH form (ΔG = -68.2 kcal/mole) than in the BH form (ΔG = -65.3), yielding a ΔΔG_BH-EH _of 2.9 kcal/mole. The bulge and loop mutations that we introduced in TAR^m ^to prevent Tat trans-activation stabilize the BH form and destabilize the EH structure. As a result the ΔΔG_BH-EH _is reduced to -3.6 kcal/mole. The most frequent +63^A-G ^substitution does not affect the stability of the BH structure but partially restores the stability of the EH form, resulting in a ΔΔG_BH-EH _of -1.2 kcal/mole. Most of the other nucleotide substitutions reduce the stability of the BH structure and at the same time stabilize the EH structure. As a result, the ΔΔG_BH-EH _of these TAR elements is increased to values between -2.0 to 6.3 kcal/mole. In cultures 4, 9 and 10, the virus accumulated multiple TAR mutations that resulted in a gradual increase in the ΔΔG_BH-EH_. In cultures 1 and 5, such a gradual increase through the accumulating mutations is not observed, but the virus acquired additional mutations in the Sp1 region. These results suggest that the bulge and loop mutations that we introduced in SIV-rtTA shifted the BH-EH equilibrium into the direction of the BH form, and that nucleotide substitutions selected during virus evolution reduce this preference for the BH form or even restore the preference for the EH structure. The only exceptions are the +46^C-T ^and +72^G-A ^mutations observed in culture 12, which only marginally affect the BH and EH stability. The virus in this culture did however acquire an additional nucleotide substitution in the Sp1 sites, which may have improved replication.

**Table 1:** Nucleotide substitutions affect the stability of the branched hairpin (BH) and extended hairpin (EH) conformation of TAR.

	Δ*G*_*BH*_^*a*^	Δ*G*_*EH*_^*a*^	ΔΔ*G*_*BH*-*EH*_^*b*^	*culture*^*c*^
TAR^wt ^(SIVmac239)	-65.3	-68.2	2.9	
TAR^m ^(SIV-rtTA)	-67.5	-63.9	-3.6	
+63A-G	-67.5	-66.3	-1.2	1^72^, 2^81^, 3^115^, 4^81^, 6^124^, 7^136^, 13^28^
+63A-G +44C-T	-65.9	-63.9	-2.0	1^153^
+63A-G +21G-A	-62.5	-66.6	4.1	4^234^
+78C-T	-65.8	-65.7	-0.1	5^32^, 10^37^
+78C-T +47T-C	-63.7	-64.1	0.4	5^198^
+21G-A	-62.5	-64.2	1.7	9^48^
+21G-A +78C-T +99C-T	-58.4	-63.6	5.2	5^124^
+21G-A +74G-A	-58.8	-63.4	4.6	9^130^
+5G-A +59A-T	-63.3	-69.6	6.3	10^136^
+73T-C	-66.5	-65.1	-1.4	11^147^
+73T-C +49C-T	-64.2	-64.9	0.7	8^222^
+46C-T +72G-A	-67.6	-63.6	-4.0	12^120^

^a ^ΔG values (kcal/mole) as determined with the Mfold RNA analysis software. ^b ^ΔΔG_BH-EH _= ΔG_BH_-ΔG_EH_. ^c ^Culture in which the mutation is observed (see Figure 2), with the day of earliest detection in superscript.

**Figure 3:**
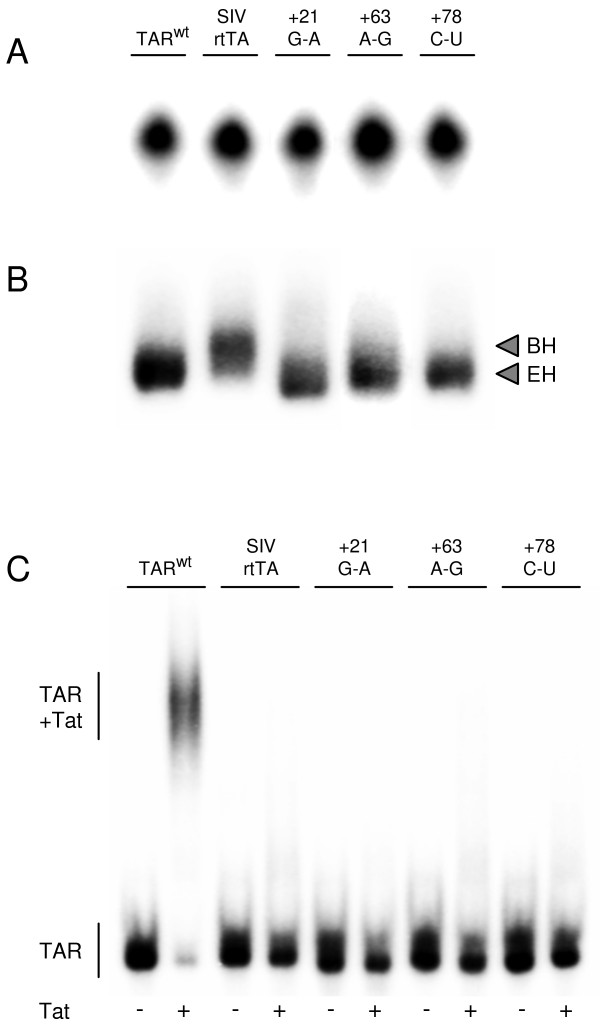
**Acquired mutations in TAR restore secondary structure but not Tat binding**. In vitro transcribed TAR RNA corresponding to the wild-type SIVmac239 (TAR^wt^), SIV-rtTA (TAR^m^) and the evolved +21^G-A^, +63^A-G ^and +78^C-U ^variants was denatured by heat, renatured in the presence of MgCl_2 _and analyzed on a denaturing gel (A) and on a non-denaturing gel (B). Under these non-denaturing conditions, branched hairpin (BH) RNA conformers migrate slower than extended hairpin (EH) molecules [28]. (C) Binding of SIV Tat to TAR was analyzed in an Electrophoretic Mobility Shift Assay (EMSA). TAR RNA was incubated with 0 or 100 ng Tat protein (indicated with - and +, respectively) and analyzed on a non-denaturing gel. The position of unbound TAR RNA and TAR-Tat complex is indicated.

To demonstrate that the introduced and acquired mutations do indeed affect TAR folding, we analyzed the electrophoretic mobility of in vitro transcribed RNAs corresponding to TAR^wt^, TAR^m ^and the evolved +21^G-A^, +63^A-G ^and +78^C-U ^variants. The RNAs were denatured by heat, renatured in the presence of MgCl_2 _and subsequently analyzed by denaturing and non-denaturing polyacrylamide gel electrophoresis. All RNAs migrate similarly on a denaturing polyacrylamide gel, as expected based on their identical size (Fig. 3A). In contrast, TAR^m ^migrates slower than TAR^wt ^on the non-denaturing gel (Fig. 3B). Since branched RNA conformers migrate slower than extended molecules, the observed migration pattern is in agreement with a predominant EH structure of TAR^wt ^under these conditions, as previously shown by Pachulska-Wieczorek et al. [28], and a BH structure of TAR^m^. The +21^G-A^, +63^A-G ^and +78^C-U ^TAR RNAs show the fast wild-type migration capacity, which demonstrates that these mutations restore EH folding of TAR in this in vitro assay.

SIV-rtTA expresses the wild-type Tat protein but the mutations introduced in TAR prevent binding of Tat and activation of transcription [25]. One possibility is that the acquired TAR mutations restore Tat binding. We therefore performed an Electrophoretic Mobility Shift Assay (EMSA) to analyze the effect of the +21^G-A^, +63^A-G ^and +78^C-U ^changes on Tat binding. In the absence of Tat, all in vitro transcribed TAR RNAs migrate similarly on the EMSA gel (Fig. 3C). Upon incubation with Tat, TAR^wt ^efficiently shifts into a slower migrating Tat-TAR complex. This Tat-TAR complex is not observed for TAR^m^, demonstrating that the introduced TAR mutations do effectively prevent Tat binding. The +21^G-A^, +63^A-G ^and +78^C-U ^substitutions do not restore Tat binding.

### Mutations in U3 and TAR do not affect promoter activity

In addition to the mutations in TAR, SIV-rtTA acquired mutations in the U3 region upon long-term culturing (Fig. 2). We observed a G-to-A substitution in one of the four G-rich Sp1 sites in six cultures. Furthermore, a 1-nt deletion in one of the Sp1 sites and a 14-nt deletion that affects two Sp1 sites were observed once. Since the U3 and TAR mutations may affect SIV-rtTA promoter activity, we re-cloned the evolved LTR sequences into an LTR promoter-luciferase reporter construct. We made constructs with the +21^G-A^, +63^A-G ^or +78^C-U ^TAR mutation. The +63^A-G ^mutation was also combined with the G-to-A substitution (mSp1) or 14-nt deletion in the Sp1 sites (ΔSp1), exactly as it appeared at day 115 in culture 3 and at day 104 in culture 1, respectively.

**Figure 4:**
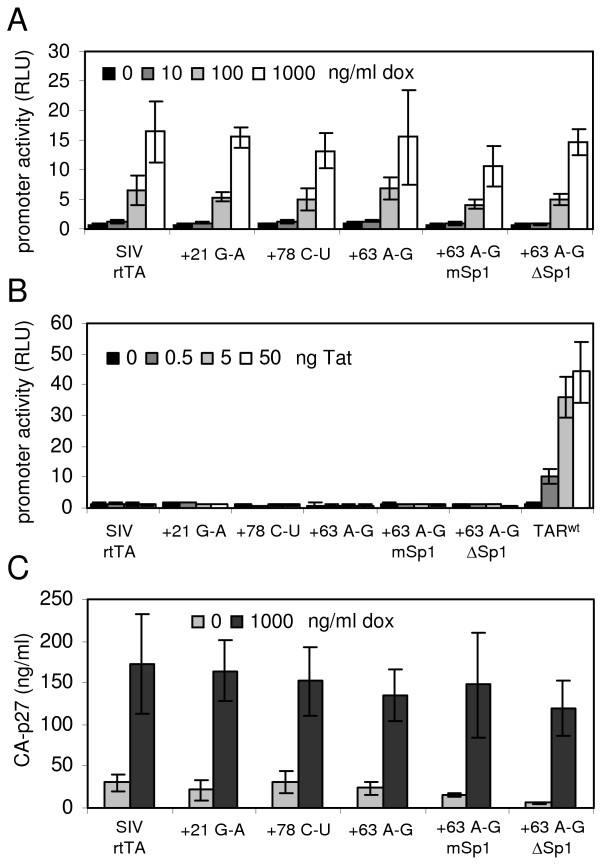
**U3 and TAR mutations do not affect dox and Tat responsiveness of the SIV-rtTA promoter**. (A) To assay dox responsiveness, C33A cells were transfected with LTR-promoter/luciferase reporter constructs corresponding to the original and evolved SIV-rtTA variants and an rtTA-expressing plasmid. After two days of culturing with 0 to 1000 ng/ml dox, the intracellular luciferase level, which reflects promoter activity, was measured. The error bar represents the standard deviation (SD) for 3 to 8 experiments (B) To assay Tat responsiveness, C33A cells were transfected with the promoter/luciferase plasmids and 0 to 50 ng SIV Tat-expressing plasmid. Two days after transfection, the promoter activity was analyzed by measuring the intracellular luciferase activity. The error bar represents the SD for 2 to 4 experiments. (C) 293T cells were transfected with the SIV-rtTA proviral constructs and cultured for two days with or without dox. Virus production was quantified by measuring the CA-p27 level in the culture supernatant. The error bar represents the standard deviation for 2 experiments.

To test the dox responsiveness of these SIV-rtTA promoters, these plasmids were co-transfected with an rtTA-expressing plasmid into C33A cervix carcinoma cells. After two days of culturing with 0 to 1000 ng/ml dox, we measured the intracellular luciferase level, which reflects gene expression (Fig. 4A). The original SIV-rtTA promoter was inactive in the absence of dox and its activity gradually increased with an increasing dox level. All evolved promoter variants showed a similar low activity without dox and a similarly high activity with dox, which demonstrates that the acquired U3 and TAR mutations do not significantly affect the basal and dox-induced promoter activity.

To test the Tat responsiveness of the new SIV-rtTA promoters, we transfected C33A cells with the promoter/luciferase plasmids plus 0 to 50 ng SIV Tat-expressing plasmid [25] and measured the luciferase level after two days (Fig. 4B). Neither the original SIV-rtTA construct nor the evolved variants responded to Tat. Only the control construct with a wild-type SIVmac239 TAR sequence showed increased activity with an increasing amount of Tat. Thus, the acquired U3 and TAR mutations do also not restore Tat responsiveness, which is in agreement with the inability of the evolved TAR RNAs to bind Tat (Fig. 3C).

### Evolved U3 and TAR sequences improve SIV-rtTA replication

To determine the effect of the acquired U3 and TAR mutations on virus production and replication, we introduced the evolved LTR sequences into the SIV-rtTA genome. The mutations were introduced in both the 5' and 3' LTR of the SIV-rtTA plasmid, such that they are stably inherited in the viral progeny. The SIV-rtTA constructs were transfected into 293T cells and after two days of culturing with or without dox, virus production was quantified by measuring the CA-p27 level in the culture supernatant (Fig. 4C). The original and new SIV-rtTA variants showed a similarly high level of virus production with dox and a similarly low level without dox. These results demonstrate that the acquired U3 and TAR mutations do not significantly affect dox-dependent viral gene expression and virus production, which is in agreement with the results of the promoter activity assays (Fig. 4A).

**Figure 5:**
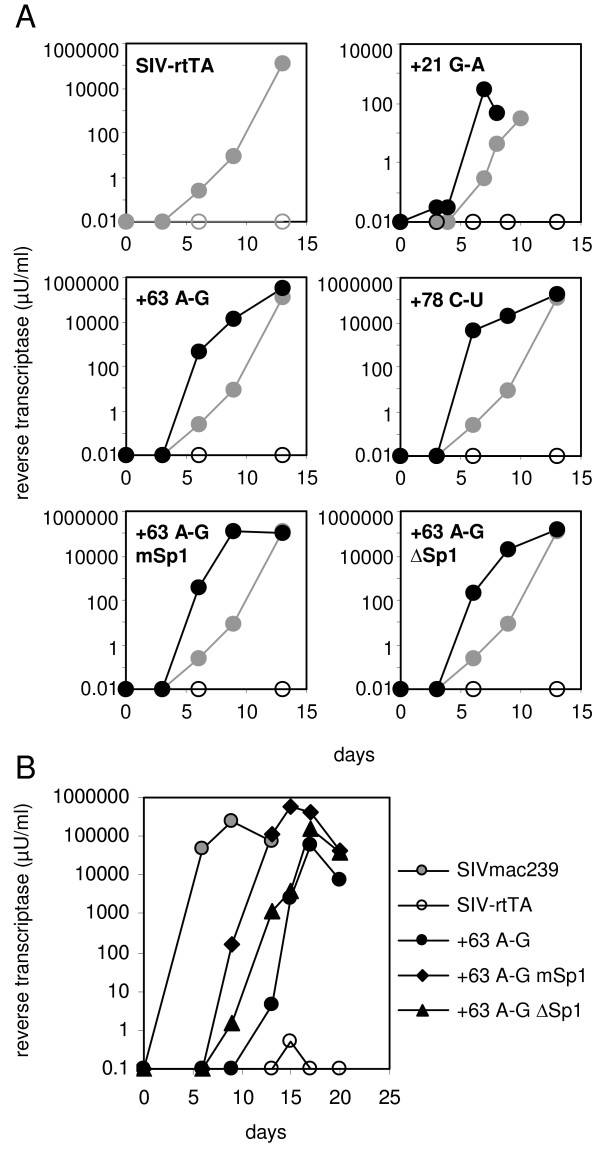
**U3 and TAR mutations improve SIV-rtTA replication**. (A) PM1 T-cells were transfected with 5 μg of the original (grey symbols) or LTR-mutated SIV-rtTA proviral plasmid (black symbols) and cultured with or without dox (closed and open symbols, respectively). Virus replication was monitored by measuring the reverse transcriptase level in the culture supernatant. (B) Cells were transfected with 1 μg SIV-rtTA or SIVmac239 proviral plasmid and cultured with dox (SIV-rtTA variants) or without dox (SIVmac239).

To evaluate the replication capacity of the SIV-rtTA variants, PM1 T-cells were transfected with 5 μg of the proviral plasmids and cultured in the presence and absence of dox (Fig. 5A). None of the SIV-rtTA variants replicate in the absence of dox, which is in agreement with their dox-dependent promoter activity. In the presence of dox, the new variants with either the +21^G-A^, +63^A-G ^or +78^C-U ^TAR mutation replicate more efficiently than the original SIV-rtTA, which demonstrates that these TAR mutations significantly improve viral replication. The +63^A-G ^mSp1 and +63^A-G ^ΔSp1 variants seem to replicate with a similar efficiency as the +63^A-G ^variant. However, comparison of the replication capacity of these variants upon transfection of 1 μg of the proviral plasmids revealed that the Sp1-mutated variants replicate more efficiently (Fig. 5B). This result demonstrates that the acquired Sp1 mutations further improve SIV-rtTA replication. The original SIV-rtTA did not show any replication within the time frame of this experiment, which illustrates that the replication capacity of the new variants has increased significantly. Despite this large improvement, the new SIV-rtTA variants did not replicate as efficiently as wild-type SIVmac239, which was included in this experiment for comparison.

**Figure 6:**
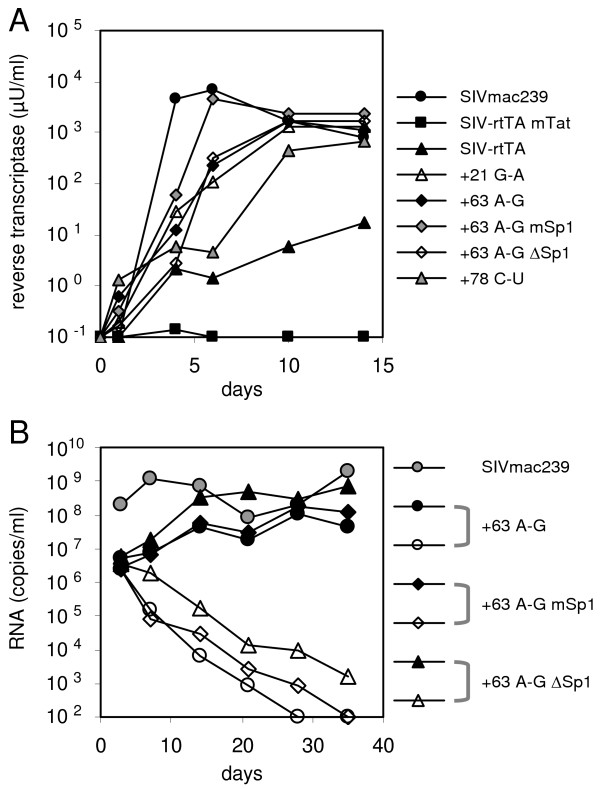
**Novel SIV-rtTA variants replicate efficiently in primary macaque PBMC**. (A) PBMC isolated from cynomolgus macaques were infected with the original or LTR-mutated SIV-rtTA variants. For comparison, cells were infected with SIVmac239. Furthermore, we included the SIV-rtTA-mTat variant in which Tat had been mutated [25]. Cells were infected with an equal amount of virus (corresponding to 10 ng CA-p27) for 16 h, washed and cultured with dox. Replication was monitored by measuring the reverse transcriptase level in the culture supernatant (B) PBMC isolated from rhesus macaques were infected with the indicated SIV-rtTA variants and SIVmac239, using comparable infectious titers (based on titration in TZM-bl cells). Cells were inoculated in the presence of dox and the cultures were split seven days later with half of the cells continuing to receive dox (closed symbols) and the other half receiving no further dox treatment (open symbols). Fresh, uninfected anti-CD3 stimulated cells from allogeneic macaque donors were added every two weeks. Replication was monitored by measuring the viral RNA copy number in the culture supernatant.

To demonstrate that the acquired mutations do not selectively improve viral replication in the human PM1 T cells that were used in the evolution study, we next assessed the replication capacity of the SIV-rtTA variants in primary PBMC isolated from cynomolgus macaques (Fig. 6A). For comparison, we included the wild-type SIVmac239 and the SIV-rtTA-mTat variant in which Tat is inactivated by a Tyr-55-Ala mutation [25]. Upon infection, cells were cultured with or without dox. In the absence of dox, none of the SIV-rtTA variants showed any replication, while SIVmac239 replicates efficiently (not shown). SIV-rtTA-mTat does also not show any replication in the presence of dox, which is in agreement with previous observations in T cell lines and indicates that SIV-rtTA requires Tat for a non-transcriptional function in the viral life cycle. The original Tat-positive SIV-rtTA replicates poorly in the PBMC upon dox administration, whereas the new variants in which we introduced the U3 and TAR changes replicate much more efficiently. However, these viruses do not replicate as efficiently as wild-type SIVmac239. Similar results were obtained when replication of the +63^A-G^, +63^A-G ^mSp1 and +63^A-G ^ΔSp1 variants was tested in PBMC isolated from rhesus macaques (Fig. 6B). Also in these cells, the new SIV-rtTA variants replicated to much higher levels in the presence of dox than in its absence, although with somewhat delayed replication kinetics when compared to SIVmac239. These studies suggest that the evolved LTR sequences significantly improve SIV-rtTA replication in macaque PBMC. Importantly, the Sp1 and TAR mutations do not affect dox-control in these primary cells.

## Discussion

In this paper, the optimization of the conditionally live SIV-rtTA variant through viral evolution is described. We recently constructed this dox-dependent SIVmac239 variant by replacing the natural Tat-TAR mechanism of transcription control by the dox-inducible Tet-On regulatory mechanism. Although the original SIV-rtTA variant replicates in T cell lines and in primary macaque PBMC, it replicates poorly when compared with the parental SIVmac239 [25](Figs. 5 and 6). Upon long-term culturing, the virus acquired several mutations in the TAR and U3 region. These mutations significantly improve viral replication, but do not affect dox control. We thus generated novel SIV-rtTA variants that replicate efficiently and in a dox-dependent manner in both T-cell lines and primary macaque PBMC.

We previously used virus evolution to optimize a similarly constructed dox-dependent HIV-1 variant. Upon long-term culturing, this HIV-rtTA variant acquired several mutations in the rtTA and tetO components of the introduced Tet-On system, which considerably improved viral replication [17-19,21]. These optimized rtTA and tetO components were used for the construction of SIV-rtTA and these elements were stably maintained upon evolution of this virus. Unlike HIV-rtTA, SIV-rtTA further improved its replication capacity through additional mutations in the TAR and Sp1 region.

For the construction of SIV-rtTA, both the bulge and loop domains in TAR were mutated to prevent binding of Tat and trans-activation of transcription. Interestingly, the acquired nucleotide substitutions in TAR upon SIV-rtTA evolution do not restore the wild-type bulge and loop sequences. The frequently observed changes at positions +63 and +78 do however allow the formation of a 6-bp spacer between the bulge and loop domains in SL2 (Fig. 1C). This is remarkable since trans-activation by HIV-2 Tat, which is very similar to SIV Tat, is optimal with a bulge-loop spacing of 6 bp [29]. However, we demonstrate that the evolved TAR elements do not bind Tat and that transcription from the modified SIV-rtTA promoter is not activated by Tat. We also frequently observed a G-to-A nucleotide substitution at position +21, which creates a 7-nt repeat at the start of SL1 and SL2. If this sequence would bind a transcription factor, either as LTR DNA or TAR RNA, duplication of the motif could increase promoter activity. However, the +21 substitution did not affect the low basal promoter activity in the absence of dox or the high induced activity in the presence of dox. Similarly, the other TAR and U3 mutations do not affect the transcription process.

In silico RNA folding analysis and in vitro RNA mobility assays revealed that the acquired TAR mutations do affect the structure of this RNA element. As previously proposed for HIV-2 TAR [28], the TAR motif of SIVmac239 and SIV-rtTA may fold alternative structures: the classical branched hairpin (BH) structure with SL1, SL2 and SL3 (Fig. 1B) and an extended hairpin (EH) structure in which the SL1 and SL2 sequences form a single, extended stem-loop structure (Fig. 1D). We demonstrate that the wild-type TAR in SIVmac239 favors the EH form. The bulge and loop mutations that we had introduced in SIV-rtTA shift the equilibrium toward the BH form. Interestingly, nearly all mutations observed in the evolved variants partially or completely restored the wild type situation in which the EH form is favored. Although the role of the EH TAR conformation and the possible EH-BH riboswitch in the SIV life cycle has yet to be resolved, these results suggest that a proper EH-BH equilibrium is important for efficient viral replication.

Interestingly, alternative folding of the leader RNA has also been proposed for HIV-1. In this case however, the TAR structure is identical in the alternative conformations. The energetically favored structure of the HIV-1 leader is formed by a long-distance interaction (LDI) between the sequences around the polyadenylation site and the dimerization initiation signal (DIS) [30]. In the alternative structure, termed the branched multiple hairpin (BMH) conformation, both the polyadenylation and DIS motifs fold a stem-loop element. Mutations that affect the equilibrium between the dimerization-incompetent LDI structure and the dimerization-prone BMH structure significantly affect HIV-1 replication [30-33]. Our recent studies with HIV-rtTA showed that HIV-1 TAR can be truncated, deleted or replaced by a non-related stem-loop element when not required for the activation of transcription, which demonstrates that TAR has no additional essential role in HIV-1 replication [34]. However, destabilization of TAR blocked replication, which can possibly be explained by unwanted pairing of free nucleotides in the destabilized TAR structure with downstream leader sequences, thereby affecting the LDI-BMH equilibrium [35]. Thus, although TAR is not a functional domain of the LDI-BMH conformational switch in HIV-1, it can indirectly affect this function. In analogy with these HIV-1 studies, it cannot be excluded that the bulge and loop mutations introduced in SIV-rtTA caused misfolding of the leader RNA. These mutations may change the local TAR folding or generate a new sequence with complementarity to downstream sequences, which could result in an interaction between TAR and other leader domains. The additional TAR mutations in the evolved variants may prevent this interaction and thus restore viral replication. Although further analyses will be needed to understand this misfolding scenario, it is supported by our recent observation that precise truncation of structural TAR domains is compatible with SIV-rtTA replication (manuscript in preparation).

We demonstrated that SIV-rtTA requires wild-type Tat protein for replication in T-cell lines [25] and primary macaque PBMC (this study), although gene expression is controlled by the incorporated Tet-On system. These results suggest that Tat has additional functions in the SIV replication cycle in addition to its role in the activation of transcription. For this reason, the SIV-rtTA variant used in this study encodes the wild-type Tat protein. Reversion of the bulge and loop mutations in TAR, which had been introduced to prevent Tat binding and trans-activation of transcription, would restore the Tat-TAR mechanism of transcription control. However, this evolution route would require multiple nucleotide substitutions, which is not likely to occur. Indeed, we never observed restoration of the Tat-TAR axis in numerous long-term cultures of SIV-rtTA. Nevertheless, the likelihood of this unwanted evolution route can be further reduced by introducing novel mutations in Tat that would inactivate the first function (activation of transcription) but not the second function (currently unknown). However, such Tat mutations remain to be identified. Alternatively, this evolution route can be blocked by the complete or partial deletion of TAR (e.g. only SL1 and SL2), as we recently showed that the complete removal of TAR in HIV-rtTA does not significantly affect replication [34].

The optimization of SIV-rtTA through viral evolution resulted in new dox-controlled variants that replicate efficiently in the PM1 T cell line and in primary PBMC from cynomolgus and rhesus macaques. These novel SIV-rtTA variants may be good candidates to study the efficacy and safety of a conditionally live virus as AIDS vaccine in macaques. Furthermore, this virus may be an ideal tool to study the immune correlates of protection if the level and duration of replication in vivo can be stringently controlled by dox administration. Such studies may reveal crucial information needed for the design of a safe and effective HIV vaccine.

## Methods

### Viruses and cells

We previously described the construction of the SIV-rtTA plasmid encoding the dox-dependent virus that is based on the SIVmac239 isolate [36](GenBank accession number M33262) and contains the wild-type Tat gene (pSIV-rtTA-Tat^wt ^in [25]). The plasmid pSIVmac239 encodes the full-length SIVmac239 isolate [37].

Human embryonal kidney (HEK) 293T and cervical carcinoma C33A cells [38] were cultured in 2-cm^2 ^wells and transfected with 1 μg SIV-rtTA or SIVmac239 DNA by calcium phosphate precipitation, as previously described [19]. PM1 T cells [39] were cultured and transfected by electroporation [25]. PBMC were isolated from cynomolgus [40] and rhesus macaques [41] and cultured as described for the PM1 cells [25]. Cells were activated with 2 μg/ml PHA (cynomolgus macaques PBMC) or anti-CD3 monoclonal antibody (rhesus macaques PBMC) for two days prior to infection with C33A or 293T produced virus, and maintained with 100 units/ml recombinant IL-2 following infection. Cells were cultured with 1000 ng/ml dox (Sigma D-9891) when indicated. The virus level in the culture medium was determined by CA-p27 ELISA (SIV core antigen kit, Beckman Coulter), by quantification of the viral RNA copy number in the culture supernatant [42], or with a real-time PCR-based reverse transcriptase (RT) assay [43] in which AMV RT was used as standard.

### Proviral DNA analysis and cloning of evolved sequences

Virus infected cells were pelleted by centrifugation at 1,500 g for 4 min and washed with phosphate-buffered saline. DNA was solubilized by resuspending the cells in 10 mM Tris-HCl pH 8.0, 0.1 mM EDTA, 0.5% Tween 20, followed by incubation with 200 μg of proteinase K per ml at 56°C for 30 min and at 95°C for 10 min. Proviral DNA sequences were PCR amplified from total cellular DNA and the purified PCR product was subsequently sequenced with nested primers. For the analysis of the 3'-half of rtTA and the 3' LTR, we used primers tTA3 (CTGTGTCAGCAAGGCTTCTC, nucleotides 9681–9700 in pSIV-rtTA-Tat^wt^) and SIV-LTR3 (ATCGGTACCGACGTCTCGAGTGCTAGGGATTTTCCTGCTTCG, nt 10991–10959) for the amplification, and tTA4 (ACGCACTGTACGCTCTGTC, nt 9709–9727), SIV-LTR8 (AAAGGGTCCTAACAGACCA, nt 10944–10926) and SIV-LTR10 (GAAGAGGGCTTTAAGCAAGCA, nt 10832–10812) for sequencing. For the analysis of Tat-coding exon 1, we used SIV-Tat2 (GGGAACCATGGGATGAATG, nt 6255–6273) and SIV-Env4 (CCCTGTCATGTTGAATTTACAGCT, nt 7192–7169) for amplification and SIV-Tat1 (GGTAGTGGAGGTTCTGGAAGA, nt 6274–6294) for sequencing. For the analysis of Tat-coding exon 2 and the 5'-half of rtTA, we used primers SIV-LTR1 (AGTACTGCGGCCGCAGGCATGCTGGGATGTGTTTGGCAATTG, nt 8691–8712) and tTA-Rev3 (TGAAATCGAGTTTCTCCAGGCCACATATGA, nt 9921–9901) for amplification, and SIV-Env5 (GGTTTGACCTTGCTTCTTGGAT, nt 8712–8733) and tTA-Rev4 (GGAAGGCAGGTTCGGCTC, nt 9882–9865) for sequencing.

For the cloning of the evolved U3 and TAR sequences into the 5' LTR of SIV-rtTA, the proviral DNA was PCR amplified with primers SIV-LTR4 (AGCTCTAGAGCGGCCGCTGGAAGGGATTTATTACAGTGCA, nt 1–36) and SIV-LTR5 (ATGGACGTCTCGAGTCGCATGCTAGGCGCCAATCTGCTAGGGATTTTCCTGCT, nt 896–867). The PCR product was ligated into the pCR2.1-TOPO TA-cloning vector (Invitrogen). The NotI-NarI fragment of the TA-clone was subsequently used to replace the corresponding 5'-LTR fragment in SIV-rtTA. For the introduction of the U3 and TAR mutations into the 3' LTR, we amplified the proviral DNA with primers tTA3 and SIV-LTR3. The PCR product was digested with EcoRI (position 10584) and XhoI (position 10981), and used to replace the corresponding 3'-LTR fragment in pBS-3'SIV-rtTA [25]. The NheI-XhoI fragment (nt 8809–10981) of these 3'-LTR modified plasmids was subsequently used to replace the corresponding fragment in the 5'-LTR modified SIV-rtTA plasmids, which resulted in SIV-rtTA constructs with the modified U3 and TAR sequences in both LTRs.

For the introduction of the evolved U3 and TAR sequences into the SIV-rtTA LTR-promoter/luciferase^firefly^-reporter construct, the EcoRI-XhoI digested tTA3/SIV-LTR3 PCR product (as described for the construction of pBS-3'SIV-rtTA variants) was used to replace the corresponding LTR fragment in the SIV-rtTA LTR-luciferase^firefly ^plasmid [25].

We used standard molecular biology procedures for all manipulations and plasmids were propagated in either *E. coli *TOP10 (for TA-cloning; Invitrogen), DH5α (pBS-3'SIV-rtTA and LTR-luc plasmids) or Stbl4 (pSIV-rtTA-Tat^wt ^constructs; Invitrogen). All constructs were verified by sequence analysis.

### Promoter activity assay

To determine dox-responsiveness of the promoter-luciferase constructs, C33A cells were transfected with 20 ng LTR-luciferase^firefly ^plasmid, 0.4 ng rtTA-expressing plasmid pCMV-rtTA_F86Y A209T _[19], 0.5 ng pRL-CMV (Promega) and 980 ng pBluescript. This pBluescript was added as carrier DNA, and pRL-CMV, in which the expression of renilla luciferase is controlled by the CMV immediate early enhancer/promoter, was co-transfected to allow correction for differences in transfection efficiency. The cells were cultured after transfection for 48 hours with 0–1000 ng/ml dox. Cells were lysed in Passive Lysis Buffer and firefly and renilla luciferase activities were determined with the Dual-Luciferase assay (Promega). The expression of firefly and renilla luciferase was within the linear range and no squelching effects were observed. The promoter activity was calculated as the ratio of the firefly and renilla luciferase activities, and corrected for between session variation [44]. To determine Tat-responsiveness of the promoter-luciferase constructs, C33A cells were transfected with 20 ng LTR-luciferase^firefly ^plasmid, plus 0–50 ng SIVmac239 Tat-expressing plasmid pcDNA3-SIV-Tat^wt ^[25], 0–50 ng pcDNA3 (empty expression vector, total amount of Tat-expressing plasmid and pcDNA3 was kept at 50 ng), 0.5 ng pRL-CMV and 950 ng pBluescript. Cells were cultured for 48 hours and luciferase activities were subsequently measured.

### Tat binding and TAR conformer assay

^32^P-labeled TAR transcripts were produced as described previously [25]. In brief, the TAR region in SIV-rtTA LTR-luciferase plasmids was amplified by PCR with a 5' primer encoding the T7 promoter sequence directly upstream of the +1 position. The DNA products were in vitro transcribed with the MEGAshortscript T7 transcription kit (Ambion). The TAR RNA transcripts were dephosphorylated with calf intestine alkaline phosphatase and 5'-end labeled with the KinaseMax kit (Ambion) in the presence of 1 μl [γ-^32^P]-ATP. The labeled transcripts were purified on a denaturating 8% acrylamide gel.

For the Tat binding assay, ^32^P-labeled TAR RNA (200 counts/s) was denatured in 10 μl water for 1 min at 85°C followed by snap cooling on ice. After addition of 10 μl 200 mM KCl, 100 mM Tris-HCl (pH 8.0), the RNA was renaturated at room temperature for 15 min. Binding of HIS-tagged SIVmac-J5 Tat protein (obtained from the Centralised Facility for AIDS reagents at the National Institute for Biological Standards and Control, Potters Bar, UK; ARP685) was analyzed by electrophoretic mobility shift assay (EMSA) as described [25]. In brief, TAR RNA (200 counts/s) was incubated with 0 or 100 ng Tat protein and 1 μg calf liver tRNA (Roche) as competitor in 50 mM Tris-HCl (pH 8.0), 20 mM KCl, 5 mM dithiothreitol, and 0.05% Triton X-100 for 15 min at room temperature, and subsequently analyzed on a non-denaturating 4% acrylamide gel containing 45 mM Tris, 45 mM Borate and 0.1% Triton X-100 at 450 V at 4°C. The gel was subsequently dried and analyzed with a PhosphorImager (Molecular Dynamics).

For the TAR conformer assay, ^32^P-labeled TAR RNA (200 counts/s) was denatured in 10 μl water for 1 min at 85°C followed by snap cooling on ice. After addition of 10 μl 200 mM KCl, 100 mM Tris-HCl (pH 8.0), 5 mM MgCl_2_, TAR RNA was renaturated at room temperature for 15 min. After adding 4 μl of non-denaturing loading buffer (30% glycerol, bromophenol blue), 10 μl of the sample was analyzed on a non-denaturating 8% acrylamide gel (in 45 mM Tris, 45 mM Borate and 0.1% Triton X-100) at 450 V at 4°C, or on a denaturing 4% acrylamide gel (in 45 mM Tris, 45 mM Borate and 0.5 mM EDTA) at 450 V at room temperature. The gels were dried and analyzed with a PhosphorImager. The thermodynamic stability of TAR RNA (nt +1 to +124) was determined with the MFold RNA folding program (version 3.2) at the Rensselaer Polytechnic Institute bioinformatics web server [26,27].

## Competing interests

The authors declare that they have no competing interests.

## Authors' contributions

ATD and BB designed the viral replication and evolution studies and drafted the manuscript; BK and AH performed the replication, evolution and promoter analysis experiments; MC and MO carried out the Tat binding and TAR conformer assays; BK, MPa, NA, FY, MPi and JL performed the PBMC experiments. All authors have read, revised and approved the manuscript.
